# Consumption of Alcohol, Cannabis, and Tobacco in a Cohort of Adolescents before and during COVID-19 Confinement

**DOI:** 10.3390/ijerph18157849

**Published:** 2021-07-24

**Authors:** Judit Rogés, Marina Bosque-Prous, Joan Colom, Cinta Folch, Tivy Barón-Garcia, Helena González-Casals, Esteve Fernández, Albert Espelt

**Affiliations:** 1 Department of Public Health, Faculty of Health Sciences of Manresa, Universitat de Vic—Universitat Central de Catalunya (UVic-UCC), Av. Universitària 46, 08242 Manresa, Spain; jroges@umanresa.cat (J.R.); sbaron@umanresa.cat (T.B.-G.); hgonzalez@umanresa.cat (H.G.-C.); aespelt@umanresa.cat (A.E.); 2 Faculty of Health Sciences, Universitat Oberta de Catalunya, Rambla del Poblenou, 156, 08018 Barcelona, Spain; 3 Departament de Psicobiologia i Metodologia en Ciències de la Salut, Universitat Autònoma de Barcelona (UAB), C/de Ca n’Altayó s/n, 08193 Bellaterra, Spain; 4 Subdirecció General de Drogodependències, Agència de Salut Pública de Catalunya, 08005 Barcelona, Spain; joan.colom@gencat.cat; 5 Centre d’Estudis Epidemiològics sobre les Infeccions de Transmissió Sexual i Sida de Catalunya (CEEISCAT), Agència de Salut Pública de Catalunya, 08916 Badalona, Spain; cfolch@iconcologia.net; 6 Centro de Investigación Biomédica en Red de Epidemiología y Salud Pública (CIBERESP), C/Monforte de Lemos 3 Pabellón 11, 28029 Madrid, Spain; 7 Tobacco Control Unit, WHO Collaborating Center for Tobacco Control, Institut Català d’Oncologia-ICO, 08908 Barcelona, Spain; efernandez@iconcologia.net; 8 Tobacco Control Research Group, Epidemiology and Public Health Programme (EPIBELL), Institut d’Investigació Biomèdica de Bellvitge-IDIBELL, 08908 Barcelona, Spain; 9 School of Medicine and Health Sciences, Campus of Bellvitge, Universitat de Barcelona, 08907 Barcelona, Spain; 10 Consortium of Centers for Biomedical Research on Respiratory Diseases (CIBERES), 20029 Madrid, Spain

**Keywords:** COVID-19, adolescents, risky substance use, lockdown, social isolation

## Abstract

The aim of this study was to identify changes in the hazardous consumption of alcohol, tobacco, and cannabis, due to the COVID-19 lockdown in 2020 in a cohort of schooled adolescents from Central Catalonia. We also analyzed the effect of the individual and social factors on risky consumption during confinement. This longitudinal study involved a subsample of 303 adolescents aged 14–18 years, who were attending 4th year of compulsory secondary education (ESO), 2nd year of college preparation (baccalaureate), or Vocational and Educational Training (VET). We collected data before COVID-19 lockdown (October 2019–February 2020) and 2 months after the lockdown ended. We estimated the prevalence of risky substance use in the sample at baseline for each independent variable. We used Poisson regression models with robust variance to compute the Cumulative Incidence (CI) and Relative Risk (RR), with their respective 95% confidence interval. We found that VET students had a significantly (*p* < 0.05) higher risk of substance use: binge drinking (RR = 3.21 (95%CI: 1.00–10.34)); hazardous drinking of alcohol (RR = 3.75 (95%CI: 1.12–12.54)), hazardous consumption of cannabis (RR = 3.75 (95%CI: 0.65–21.59)) and daily smoking of tobacco (RR = 4.82 (95%CI: 1.74–13.39)). The results showed a general trend of reduction of consumption during COVID-19 confinement period. This study suggests that VET students were more likely to engage in hazardous drinking of alcohol and daily smoking of tobacco. No statistically significant differences were found for the other age groups and variables.

## 1. Introduction

In March 2020, the World Health Organization (WHO) declared a global pandemic situation. In order to control the COVID-19 outbreak and minimize its spread, social contact was reduced through restrictive measures, such as periods of lockdown [1,2,3,4,5,6]. Currently, the possible long-term repercussions of the COVID-19 confinements are unknown. However, research on previous SARS-CoV outbreaks suggests that social distancing, and lockdown periods are potentially stressful situations for the adult population. Therefore, these periods could contribute to increase the consumption of substances and trigger abuse and dependence [7,8]. Moreover, previous studies addressed the negative effects of confinements on health among the general population and claim that the derived psychological and emotional distress, like anxiety, depression, stress, boredom, and loneliness, can lead to risky consumption [3,4,5,6,9]. Indeed, people might consume risky substances to cope with or seek relief from the mentioned unpleasant emotions [3,4,5,6,8,10].

Adolescence is a period of change and transition to adulthood when the initiation into substance use is common [11]. With age, this consumption could gradually increase in frequency and intensity, very likely triggering a dependence [12]. During adolescence, the consumption mainly occurs in contexts of socialization with peers, away from the parental control [12,13,14]. Some studies showed that confinement forced adolescents to spend less time in social contexts and more with parents. This could have increased the parental supervision and interfered with the access to substances, leading to a lower consumption [1,2,4,6,12,15]. On the contrary, some authors suggested that confinement resulted in alterations of the routine and lifestyle, and this might have led teenagers to assume unhealthy habits, including an increased consumption of substances like alcohol and tobacco [3,4,8]. In this regard, the consumption of legal and illegal substances by adolescents during the COVID-19 lockdown periods took place in four contexts: (a) infringing the lockdown restrictions and meeting with peers; (b) engaging in lone risky consumption; (c) increasing their substance use with parents or other family members; and, finally, (d) using social networks to simultaneously consume risky substances with peers [1,6,16].

Most data, however, derive from cross-sectional studies and targeted adults. Therefore, it is interesting to know how the patterns of substance use changed during lockdown periods in adolescents, through longitudinal studies. The aim of this project was to identify the changes in binge drinking, the hazardous drinking the hazardous consumption of cannabis and the daily smoking of tobacco, in a cohort of 14 to 18-year-old adolescents, due to the COVID-19 pandemic confinement in Central Catalonia. We also analyzed the differences in risky consumption and the effect of individual and contextual factors in this consumption during confinement.

## 2. Materials and Methods

### 2.1. Setting and Sample

This is a longitudinal study that was part of the DESK-Cohort project (http://deskcohort.cat/) (accessed on 22 July 2021), with a subsample of high-schooled adolescents aged 14–18 years from Central Catalonia. The students were attending the 4th year of compulsory secondary education (ESO), the 2nd year of college preparation (baccalaureate), or the Vocational and Educational Training (VET), during the 2019–2020 academic course. The DESK-COVID-Cohort project arises in the context of COVID-19 with the aim to follow-up the sample of adolescents surveyed in pre-pandemic period and to know the effects of the confinement on health-related behaviors and substance use in those adolescents who were part of the cohort.

### 2.2. DESK-COVID-Cohort Questionnaires

The DESK-COVID-Cohort survey collected data about health-related behaviors, such as: sleep quality; mood; dietary habits; physical activity; use of screens and new technologies; and consumption of alcohol, cannabis, and tobacco. The first wave of the DESK-COVID-Cohort study took place between October 2019 and February 2020. In this wave the subsample was formed by 1442 students over 14 years old who were attending the 4th year of compulsory secondary education, the 2nd year of college preparation, or the Vocational and Educational Training. At this time, the questionnaire was self-reported and data were collected in schools by using a tablet connected to a Research Electronic Data Capture (Redcap) system [17] following the Organic Law on Protection of Personal Data regulation. To ensure anonymity and monitoring the participants for further waves, a code with initial name and surname letters was created automatically for each participant. All students and their families accepted to participate in the project and signed the informed consent before the study started. Finally, the study was approved by the Ethical Committee of the Vic University–Central University of Catalonia (127/2020). Furthermore, to preserve anonymity and monitoring the participants for further waves, in the questionnaire, each participant was requested to indicate the two initial name and surname letters. A code was created automatically for each participant. The numeric code allows to link data of the same participants in the different waves. Only the principal investigator has access to this database.

In order to assess the effects of the COVID-19 confinement on health-related behaviors, we developed the DESK-COVID-Cohort questionnaire, a reduced version of the first wave’s questionnaire, which included questions related to health behaviors and the use of risky substance during the lockdown period. Additionally, the second wave of DESK-COVID-Cohort survey added questions about the conditions of the family during confinement, the pandemic effects on different aspects of health, and the personal measures taken against the virus [18]. In the first wave of DESK-COVID-Cohort the questionnaire was self-administered in high schools using a tablet. Instead, in the second wave the questionnaire (a modified and reduced version) was sent by email or WhatsApp to the 1442 students that signed the informed consent in the first wave of the DESK-COVID-Cohort, accepting to participate in further studies. A subsample of 303 adolescents who were attending the 4th year of ESO, 2nd year of baccalaureate, and VET, participated in this study.

The first wave of the DESK-COVID-Cohort was conducted from October 2019 to February 2020, before the pandemic started in Catalonia (the first case was diagnosed on 25 February 2020) [19] and for the second wave data were collected from June to July 2020, after the period of lockdown in Catalonia.

**Figure 1:**
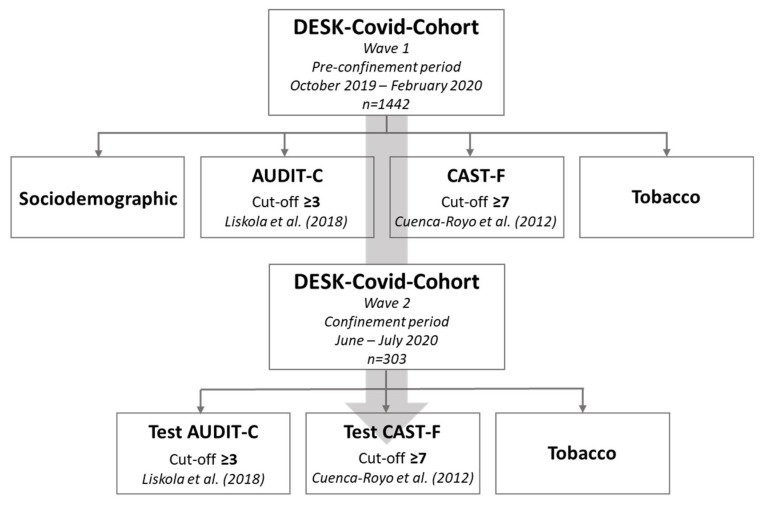
Methodology of DESK-COVID-Cohort in the two waves of the study and tests used to assess substance use.

Figure 1 describes the process followed for data collection, specifying the type of data collected in the first and second wave of DESK-COVID-Cohort questionnaires, the period in which data were collected and the participants in each wave. Additionally, it includes information about the tools used to evaluate the binge drinking, hazardous drinking, hazardous consumption of cannabis, and daily smoking of tobacco.

### 2.3. Measurements

The main dependent variable was “worsening the consumption of alcohol, cannabis, or tobacco”, which was created comparing substance use previously to the lockdown period (data from first wave of DESK-COVID-Cohort questionnaire) and during that period (data from second wave of DESK-COVID-Cohort questionnaire). The increase or maintenance of the prevalence of binge drinking, hazardous drinking, hazardous consumption of cannabis, or daily smoking of tobacco during the period of confinement due to the COVID-19 pandemic was considered as worsening substance use because ongoing use and risky use during the period of confinement was accounted for as detrimental for health.

Alcohol consumption was measured through the AUDIT-C test (Alcohol Use Disorders Identification Test), which is a validated screening tool for hazardous drinking and alcohol dependence. The binge drinking variable was created from the answers to the question “How often do you have ≥6 alcoholic drinks on a single occasion?”. An occasion was defined as the act of drinking in a row, or in an interval of approximately 2–4 h. Participants’ responses were divided into two categories (“binge drinking” and “no binge drinking”). Only those subjects who indicated the option “never” were grouped in the “no binge drinking” category. To measure hazardous drinking, we used the three questions from the AUDIT-C test, which were related to frequency and quantity of alcohol consumption, and frequency of binge drinking. Responses to each question were added up, and a final score from 0 to 12 was obtained. A cut-off of ≥3 points indicates hazardous alcohol consumption, according to criteria established by Liskola et al. in 2018 [20].

Hazardous consumption of cannabis was measured using the CAST validated test (Cannabis Abuse Screening Test), which measures the frequency of events related to cannabis consumption in the 12 months before the study. However, in the second wave of the DESK-COVID-Cohort survey, the questions focused on the period of confinement. Participants with a score ≥7 were included in the risky consumption group [21].

To assess daily smoking of tobacco, we asked the question “How often have you smoked tobacco?”. Participants were divided in two categories (“daily smoking-tobacco” and “no daily smoking-tobacco”). Those subjects who indicated the option “every day” were considered daily smokers and the rest were grouped in the “no daily smoking-tobacco” category.

The main independent variable was the data collection period (pre-COVID-19 versus COVID-19 lockdown). We also compared the prevalence of consumption during the COVID-19 lockdown period among different groups of the following individual and contextual factors: course of the participants (4th year of compulsory secondary education (ESO) (15–16 years old), 2nd year of college preparation (baccalaureate) (17–18 years old), or Vocational and Educational Training (VET) (16–17 years old)); municipality of residence (rural (≤10,000 inhabitants) or urban (>10,000 inhabitants)); impact of COVID-19 measures on the region (higher impact with earlier and stricter lockdown measures (Anoia) or lower impact (other regions of Central Catalonia)); level of education attained by parents (primary, secondary, or university); and self-reported socioeconomic position (SEP) (participants were divided in terciles: lower SEP, medium SEP, or higher SEP) [22].

### 2.4. Statistical Analysis

First, we described the baseline characteristics of the sample in the first and second wave. Then, we calculated the proportion of the adolescents engaging in binge drinking, hazardous drinking, hazardous consumption of cannabis, and daily use of tobacco, with 95% confidence interval (95%CI), in both waves of the DESK-COVID-Cohort. Last, we estimated Poisson regression models with robust variance [23]. This way, we obtained the Cumulative Incidence of Change (IC) and the relative risks (RR) of the worsening consumption of each substance among subpopulations stratified according to the different contextual and individual factors, with 95%CI. All the analyses were conducted using STATA 16 (StataCorp, College Station, TX, USA). 

## 3. Results

Table 1 describes the characteristics of the participants in the two waves of the DESK-COVID-cohort. The sample in first wave was formed by 1442 participants (43.3% boys and 56.7% girls). In the second wave, the sample was formed by 303 participants (29.7% boys and 70.3% girls), 55.8% of which were attending 4th year of ESO, 34.3% the 2nd year of baccalaureate, and 9.9% the VET. A total of 279 participants indicated their parents’ academic level, and 43% of their parents had a university degree. Altogether, 82.2% of the participants resided in a less-affected (by COVID-19 measures) municipality of Central Catalonia, and almost two-thirds of them lived in a rural municipality. As for substance use, 36.3% of participants consumed alcohol in a binge drinking pattern, 38.9% were risky users, 4.6% were hazardous cannabis users, and 8.9% of participants reported being daily smokers.

As shown in Table 2, the overall prevalence of binge drinking decreased between pre COVID data (DESK-COVID-Cohort questionnaire, first wave) and after confinement data (DESK-COVID-Cohort questionnaire, second wave) from 36.3% to 5.9%, respectively (*p* < 0.05). The hazardous drinking of alcohol was declared by 38.9% of the cohort members before lockdown and dropped to 5.6% post lockdown (*p* < 0.05). The prevalence of hazardous consumption of cannabis decreased from 4.6% to 2.3% (*p* > 0.05); and the overall prevalence of daily smoking decreased from 8.9% to 6.3% (*p* > 0.05). On the contrary, the prevalence of daily smoking increased among specific categories, such as: VET students, participants with parents with primary education, and inhabitants of the more affected municipality of Anoia (Table 3). Despite the general decrease in consumption, we found that during confinement there was one participant that tried alcohol for the first time and another that started to smoke tobacco.

We also analyzed the differences in the prevalence of risky consumption between social groups during the lockdown period. Results are reported in Table 4 and Table 5, which includes the IC and RR of risky consumption during the COVID-19 confinement. VET students had a significantly (*p* < 0.05) higher risk of binge drinking (RR = 3.21 (95%CI: 1.00–10.34)); hazardous consuming alcohol (RR = 3.75 (95%CI: 1.12–12.54)), and daily tobacco smoking (RR = 4.82 (95%CI: 1.74–13.39)), compared to 4th year ESO and 2nd baccalaureate students. Compared to 4th year ESO students, VET students had 1.87 more risk of worsening their patterns of substance use during the COVID-19 lockdown period. Additionally, the probability to show worse patterns of substance use was higher for adolescents with parents with secondary education (RR = 1.42 (95%CI: 0.24–8.33)) or lower (RR = 2.33 (95%CI: 0.59–9.11)), than subjects with parents that had a university degree. The likelihood of binge drinking (RR = 1.46 (95%CI:0.49–4.33)) and hazardous drinking of alcohol (RR = 2.43 (95%CI: 0.66–8.95)) were higher among medium and high SEP categories (Binge drinking RR = 1.26 (95%CI:0.37–4.23); risky alcohol use RR = 2.53 (95%CI: 0.65–9.83)), in comparison to low SEP categories.

## 4. Discussion

To our knowledge, this is the first longitudinal survey that studies the changes of alcohol, cannabis, and tobacco consumption during the COVID-19 lockdown period in a cohort of teenagers from Central Catalonia. Our findings showed a general trend of reduction of binge drinking, hazardous drinking, hazardous consumption of cannabis, and daily tobacco smoking, among 14- to 18-year-old high-school students during the COVID-19 lockdown period. Despite decreasing, we must not belittle the prevalence of consumption and risky consumption.

The results of this project allowed us to establish relationships between individual and contextual factors and the changes in the use of risky substance in adolescents, due to the COVID-19 confinement. It is noteworthy that the dependent variable “worsening the risky consumption” includes both increasing and maintaining the use of substances in a risky pattern. Indeed, we considered worrying continuing to engage in risky consumption during the extreme situation of confinement, despite the stay-at-home measures and difficulties that adolescents might have faced to get some substances and consume them inside or outside home.

One limitation of this study could be that our questionnaires excluded the population out of formal educative system; therefore, the sample is representative of most students aged 14–18, but not all. Besides, in this sample we have seen that girls engage in a higher risky consumption of some substances compared to boys. These results contradict the literature, which affirms a higher risky consumption in boys. Despite this, the differences are not statistically significant, and in the analysis of the evolution of substance use during confinement, the conclusions on consumption continue to show that this pattern does not change by sex. In addition, the self-reported variables of the DESK questionnaires could have generated information biased results (for example for social desirability). However, the evidence affirms that the use of self-reported questionnaires is a reliable method to detect the alcohol and cannabis consumption in adolescents [24,25]. Another limitation might be the way the questionnaires were distributed, as those were sent by mail and WhatsApp, and these channels often have a high prevalence of no response.

As already mentioned, the COVID-19 lockdown period modified the patterns of binge drinking, the hazardous drinking, hazardous consumption of cannabis, and smoking tobacco among schooled adolescents. The lack of statistically significant differences could be due to the sample size. Despite this, a tendency of changes in substance use was found in some variables, which suggests that there are sectors of the population that have been less affected by the confinement due to the COVID-19 pandemic. Indeed, our results showed a higher prevalence and probability of engage in binge drinking, hazardous drinking, hazardous consumption of cannabis, and more daily use of tobacco in older adolescents attending advanced courses (VET students), compared to the rest of the participants of lower courses [12].

During adolescence, socialization with peers provides and facilitates opportunities for consumption; therefore, the social contact constraint and isolation due to COVID-19 made the access to risky substances more challenging [2,5,6,15]. Nevertheless, some studies suggest that situations of social distancing during the pandemic could have negative consequences on psychological and mental health [1,3,5,10,11]; therefore, some adolescents could have engaged in substance use as a way to cope or deal with all the psychological discomfort and negative feelings related to the COVID-19 situation [3,4,5,6,8,11]. Despite most adolescents live with their parents or family members, the maintenance or increase in substance use could be explained by the fact that some adolescents stayed alone at home while parents went to work because they could not do telework. So, maybe the adolescents seized the lack of parental control to use substance at home.

There are various factors that differently affect the consumption of specific substances. In this case, legality and accessibility of some substances seem to play an important role in the atypical situation of confinement. The binge drinking and the hazardous drinking take place specially in social occasions with peers, so the social distancing and the limitation of the leisure could explain the observed reduction in alcohol consumption [14]. As for cannabis, we observed a more considerable reduction and it might be because the situation of confinement could have made it difficult to obtain this illicit substance, reducing the availability and accessibility [6,15]. The decrease in tobacco smoking was less accentuated than the decrease in the consumption of other substances during the COVID-19 lockdown period. This might be because tobacco is a legal substance, easily accessible even at home and tobacco retailers did continue their activity during lockdown. A large number of studies claim that the likelihood of risky consumption is higher among rural adolescents, compared to the ones living in urban areas [26,27]. However, other studies agree with the results obtained in the present survey, which shows a greater consumption of substances in adolescents living in urban settings [28]. As it was already mentioned, maybe cities had more open establishments and people had more options to acquire substances during the lockdown period. Our study is in line with other investigations showing a connection between lower parental education and higher prevalence of consumption [28,29,30,31]. Indeed, we found that adolescents with parents with primary education were more likely to use substances than adolescents with parents with secondary and university education.

Alcohol was the most consumed substance before the lockdown, and the one that underwent the greatest reduction during the COVID-19 lockdown period. In the same direction of other studies that identified an association between the socioeconomic status and substance abuse, our findings show that adolescents with a medium or high SEP drank more frequently and with a riskier pattern, in comparison to adolescents with a low SEP, both before and after confinement [32,33,34]. Our data reveal a notable decrease in the prevalence of binge drinking and hazardous drinking during the lockdown period in all groups, objectifying the impact of restrictions and social distancing on adolescents. In agreement with other surveys, we found that younger adolescents drank less alcohol during confinement. This might be due to the closure of leisure establishments and the suspension of school classes, that prevented social meetings and reduced the opportunities to access psychoactive substances [2,35]. Furthermore, parental disapproval towards binge drinking could explain the reduction of risky consumption patterns [16].

The hazardous consumption of cannabis showed the lowest prevalence among the different substances before confinement, and its prevalence was even lower during the COVID-19 lockdown period. Under normal conditions, adolescents perceive that accessibility to cannabis is high, even though cannabis is an illegal substance [36]. However, we believe that COVID-19 confinement and the restrictions to mobility and social contact could have increased the obstacles for the illicit trade of the substance, thus reducing its availability and accessibility [6,15]. Additionally, spending more time with the family, together with parental disagreement on the use of cannabis, could have contributed to diminish its risky consumption [16]. Our results agree with previous studies showing that people with a lower SEP and those who live in urban areas have higher opportunities to consume illicit substances as cannabis [37,38,39]. Indeed, we observed a maintenance in the hazardous consumption of cannabis among people living in disadvantaged neighborhoods. We think that this might be explained by the frequent breaches of the confinement restrictions and the easy of trafficking and acquisition of the substance in these areas.

The reduction in daily smoking was the lowest in comparison to the reduction of the other substances during confinement. While some groups decreased their daily consumption (especially 4th ESO students and participants whose parents had a university education), others even increased it (VET student, participants whose parents had a primary education, and those living in Anoia). At these ages, we find a large part of social smokers, who smoke tobacco in social settings and with the group. The COVID-19 pandemic led to restrictions of social meetings, spending more time with parents at home, and in some cases the information about the worsening of respiratory problems that COVID could generate in smokers increased the health risk awareness [5], and could explain the observed decrease. Moreover, a family attitude toward the tobacco with parental disapproval towards its consumption at home could have contributed to this reduction. On the contrary, the increased consumption in some groups could be a way to alleviate negative emotions related to COVID-19, beat boredom, and overcome the lack of social relations [3,5,9,14]. Additionally, some parents may have been using tobacco to cope with the situation, making it more accessible to their sons, or maybe being more permissive and allowing them to smoke at home [6,15].

The results of this study provide the first data about the effect of COVID-19 confinement on consumption of legal and illicit substances among teenagers (14–18 years) from Central Catalonia. The measures of social distancing forced adolescents to spend more time at home with family members, and less in social and leisure environments. This reduced the opportunities of engaging in binge drinking and the consumption of alcohol, cannabis and tobacco. However, in spite of the general tendency to decrease, a higher risky consumption of substances was observed in older adolescents attending advanced courses. We believe that, by reducing risky consumption among teenagers, confinement could reduce the probability of future related problems. To confirm this hypothesis, further follow up of the cohort is warranted once the pandemic situation has passed and restrictions about mobility and social contacts had been overcome.

## 5. Conclusions

Our findings suggest that the COVID-19 situation affected the social life of young people and decreased risky consumption in most adolescents. The confinement measures during the pandemic period limited the socialization and increased the parental control and the time spend with family away from peers. In general, substance use was reduced in the confinement period, but the group of vocational training showed a higher risk to be involved in hazardous use of alcohol and cannabis, and more daily use of tobacco.

The observed changes in substance use show the influence of parental control and the role of socialization on the likelihood of engaging in risky behaviors. This highlights the importance to involve the parents in programs of prevention and perform actions in adolescents’ social environments. Likewise, the study could help to evaluate interventions and design programs aimed at improving the future health behavior of the population. Finally, knowing how confinement affected the patterns of substance use among adolescents provides useful data for public authorities to adapt preventive and health promotion activities to better target the needs of young people.

## Figures and Tables

**Table 1:** Sociodemographic characteristics and binge drinking, hazardous drinking, hazardous consumption of cannabis, and tobacco of participants in Wave 1 and Wave 2. The DESK-COVID-Cohort study.

Total	DESK-COVID-Cohort				
	Wave 1		Wave 2		*p*-Value
	Total		Total		
	*n*	%	*n*	%	
	1442	100	303	100	
Sex					
Boys	624	43.3	90	29.7	<0.001
Girls	818	56.7	213	70.3	
Course					
4th ESO	915	63.5	169	55.8	0.052
2nd baccalaureate	402	27.9	104	34.3	
VET	120	8.3	30	9.9	
Education level of parents					
University	572	44	120	43	0.83
Secondary	456	35	103	36.9	
Primary	274	21	56	20.1	
SEP ^a^					
Lower SEP	537	37.2	105	34.7	0.15
Medium SEP	464	32.2	115	38	
Higher SEP	441	30.6	83	27.4	
Municipality of residence ^b^					
Rural	830	59.3	176	60.3	0.75
Urban	570	40.7	116	39.7	
Impact of COVID-19 on the region ^c^					
More affected (Anoia)	267	18.5	54	17.8	0.78
Less affected (others)	1175	81.5	249	82.2	
Binge drinking ^d^					
Yes	480	56.7	110	36.3	<0.001
No	367	43.3	193	63.7	
Hazardous drinking (AUDIT-C)					
Yes	513	64.4	118	38.9	<0.001
No	929	35.6	185	61.1	
Hazardous consumption of cannabis (CAST)					
Yes	81	5.6	14	4.6	0.017
No	1361	94.4	289	95.4	
Daily smoking-Tobacco					
Yes	156	10.8	27	8.9	0.017
No	1286	89.2	276	91.1	

^a^ SEP = socioeconomic position. Terciles obtained from McArthur Scale results, which inquired for neighborhood self-perceived SEP; ^b^ Variable dependent on the number of inhabitants (≤10,000 rural; >10,000 urban); ^c^ Different impact on the regions of Central Catalonia. Anoia was more affected, with earlier and stricter lockdown measures, compared to the other regions; ^d^ Variable indicating the frequency of consumption of ≥6 alcoholic drinks in a single occasion (in 2–4 h). Not all the variables had the responses of the total sample.

**Table 2:** Prevalence of binge drinking and hazardous consumption of alcohol, before and during lockdown due to the COVID-19 pandemic in the 303 adolescents from Central Catalonia who participated in wave 1 and wave 2 of DESK-COVID-Cohort project.

Total	Binge Drinking				Hazardous Drinking			
	Pre		During		Pre		During	
	%	95% CI	%	95% CI	%	95% CI	%	95% CI
	36.3	(31.1–41.9)	5.9	(3.8–9.2)	38.9	(33.6–44.6)	5.6	(3.5–8.89)
Sex								
Boys	33.3	(24.4–43.7)	6.7	(3.0–14.1)	37.8	(28.4–48.2)	8.9	(4.5–16.8)
Girls	37.6	(31.3–44.3)	5.6	(3.2–9.7)	39.4	(33.1–46.2)	4.2	(2.2–7.9)
Course								
4th ESO	29.0	(22.6–36.3)	4.1	(1.9–8.5)	30.2	(23.7–37.5)	3.5	(1.6–7.78)
2nd baccalaureate	44.2	(34.9–53.9)	6.7	(3.2–13.5)	50.0	(40.5–59.5)	6.7	(3.2–13.5)
VET	50.0	(32.8–67.2)	13.3	(5.1–30.7)	50.0	(32.8–67.2)	13.3	(5.1–30.7)
Education level of parents								
University	33.3	(25.5–42.3)	3.3	(1.3–8.6)	37.5	(29.3–46.5)	5.0	(2.3–10.7)
Secondary	35.0	(26.3–44.7)	6.8	(3.3–13.6)	35.9	(27.2–45.6)	5.8	(2.6–12.4)
Primary	44.6	(32.2–57.8)	7.1	(2.7–17.6)	44.6	(32.2–57.8)	7.1	(2.7–17.6)
SEP								
Lower SEP	33.3	(24.9–42.9)	4.8	(1.9–10.9)	32.4	(24.1–41.9)	2.9	(0.9–8.5)
Medium SEP	36.5	(28.2–45.7)	7.0	(3.5–13.3)	39.1	(30.6–48.4)	7.0	(3.5–13.3)
Higher SEP	39.8	(29.8–50.6)	6.0	(2.5–13.7)	47.0	(36.5–57.7)	7.2	(3.2–15.2)
Municipality of residence								
Rural	34.7	(27.9–42.0)	4.0	(1.9–8.1)	38.1	(31.3–45.5)	4.5	(2.3–8.8)
Urban	38.8	(30.3–47.9)	8.6	(4.8–15.3)	41.4	(32.8–50.6)	6.9	(3.6–13.2)
COVID-19 impact on the region								
More affected (Anoia)	40.7	(28.5–54.2)	3.7	(0.9–13.7)	42.6	(30.2–56.0)	5.5	(1.8–15.9)
Less affected (others)	35.3	(29.6–41.5)	6.4	(3.9–10.2)	38.2	(32.3–44.4)	5.6	(3.4–9.3)

Abbreviatures: SEP = socioeconomic position. Terciles obtained from McArthur Scale results, which inquired for self-perceived SEP.

**Table 3:** Prevalence of hazardous consumption of cannabis, and daily smoking, before and during lockdown due to the COVID-19 pandemic in the 303 adolescents from Central Catalonia who participated in wave 1 and wave 2 of DESK-COVID-Cohort project.

Total	Hazardous Consumption of Cannabis				Daily Smoking of Tobacco			
	Pre		During		Pre		During	
	%	95%CI	%	95%CI	%	95%CI	%	95%CI
	4.6	(2.7–7.8)	2.3	(1.1–4.8)	8.9	(6.2–12.7)	6.3	(4.0–9.6)
Sex								
Boys	2.2	(0.6–8.5)	2.2	(0.6–8.5)	4.4	(1.7–11.3)	5.6	(2.3–12.7)
Girls	5.6	(3.2–9.7)	2.3	(1.0–5.5)	10.8	(7.3–15.7)	6.6	(3.9–10.8)
Course								
4th ESO	4.7	(2.4–9.2)	1.8	(0.6–5.4)	5.3	(2.8–9.9)	4.1	(1.9–8.5)
2nd baccalaureate	1.9	(0.5–7.4)	1.9	(0.5–7.4)	10.6	(5.9–18.1)	5.8	(2.6–12.3)
VET	13.3	(5.1–30.7)	6.7	(1.7–23.2)	12.3	(11.5–41.6)	20	(9.2–38.0)
Education level of parents								
University	2.5	(0.8–7.5)	2.5	(0.8–7.5)	5.8	(2.8–11.8)	3.3	(1.3–8.6)
Secondary	2.9	(0.9–8.7)	1.0	(0.1–6.6)	10.7	(5.9–18.3)	4.9	(2.0–11.2)
Primary	12.5	(6.2–24.0)	3.6	(0.9–13.2)	8.9	(3.8–19.8)	10.7	(4.9–21.9)
SEP								
Lower SEP	3.8	(1.4–9.7)	3.8	(1.4–9.7)	10.5	(5.9–17.9)	6.7	(3.2–13.4)
Medium SEP	5.2	(2.4–11.2)	1.7	(0.4–6.7)	7.8	(4.1–14.4)	5.2	(2.34–11.2)
Higher SEP	4.8	(1.8–12.2)	1.2	(0.2–8.1)	8.4	(4.1–16.7)	7.2	(3.3–15.2)
Municipality of residence								
Rural	3.4	(1.5–7.4)	1.7	(0.5–5.2)	6.3	(3.5–10.9)	4.5	(2.3–8.8)
Urban	6.9	(3.5–13.2)	3.4	(1.4–8.9)	12.1	(7.3–19.4)	9.5	(5.3–16.4)
COVID-19 impact on the region								
More affected (Anoia)	9.3	(3.9–20.4)	1.9	(0.3–12.2)	5.6	(1.8–15.9)	7.4	(2.8–18.2)
Less affected (others)	3.6	(1.9–6.8)	2.4	(1.1–5.3)	9.6	(6.5–13.9)	6.0	(3.7–9.8)

Abbreviatures: SEP = socioeconomic position. Terciles obtained from McArthur Scale results, which inquired for self-perceived SEP.

**Table 4:** Cumulative Incidence of worsening of binge drinking and hazardous consumption of alcohol, between groups during confinement and their associated factors in adolescents from Central Catalonia.

Total	Binge Drinking				Hazardous Drinking			
	CIw	95%CI	RR	95%CI	CIw	95%CI	RR	95%CI
	5.94	(3.76–9.24)			5.61	(3.51–8.85)		
Sex								
Boys	6.67	(3.02–14.09)	1		8.89	(4.49–16.82)	1	
Girls	5.63	(3.22–9.68)	0.85	(0.33–2.19)	4.22	(2.21–7.94)	0.48	(0.19–1.19)
Course								
4th ESO	4.14	(1.98–8.45)	1		3.55	(1.59–7.70)	1	
2nd Baccalaureate	6.73	(3.23–13.48)	1.63	(0.59–4.51)	6.73	(3.23–13.48)	1.90	(0.65–5.49)
VET	13.33	(5.07–30.68)	3.21 *	(1.0–10.35)	13.33	(5.07–30.68)	3.76*	(1.12–12.54)
Education level of parents								
University	3.33	(1.25–8.61)	1		5.00	(2.25–10.71)	1	
Secondary	6.79	(3.26–13.61)	2.14	(0.55–8.28)	5.82	(2.63–12.40)	1.43	(0.42–4.87)
Primary	7.14	(2.69–17.60)	2.04	(0.61–6.78)	7.14	(2.69–17.60)	1.17	(0.39–3.51)
SEP								
Lower SEP	4.76	(1.98–10.96)	1		2.85	(0.92–8.52)	1	
Medium SEP	6.95	(3.51–13.32)	1.46	(0.49–4.33)	6.95	(3.51–13.32)	2.43	(0.66–8.95)
Higher SEP	6.02	(2.52–13.71)	1.27	(0.38–4.23)	7.22	(3.27–15.21)	2.53	(0.65–9.83)
Municipality of residence								
Rural	3.97	(1.90–8.12)	1		4.54	(2.28–8.84)	1	
Urban	8.62	(4.68–15.31)	2.17	(0.85–5.54)	6.89	(3.47–13.22)	1.52	(0.58–3.93)
COVID-19 impact on the region								
More affected (Anoia)	3.70	(0.92–13.70)	1		5.55	(1.79–15.92)	1	
Less affected (others)	6.42	(3.96–10.24)	1.73	(0.41–7.34)	5.62	(3.35–9.28)	1.01	(0.30–3.41)

* *p* < 0.05. Abbreviatures: SEP = socioeconomic position. Terciles obtained from McArthur Scale results, which inquired for neighborhood self-perceived SEP. CIw: Cumulative Incidence of worsening of risky consumption for each substance between pre-COVID-19 and during COVID-19 confinement.

**Table 5:** Cumulative Incidence of worsening of hazardous consumption of cannabis, and daily smoking, between groups during confinement and their associated factors in adolescents from Central Catalonia.

	Hazardous Consumption of Cannabis				Daily Smoking—Tobacco				Worsening Consumption ^a^			
	CIw	95%CI	RR	95%CI	CIw	95%CI	RR	95%CI	CIw	95%CI	RR	95%CI
Total	2.31	(1.10–4.77)			6.27	(4.02–9.63)						
Sex												
Boys	2.22	(0.55–8.49)	1		5.56	(2.32–12.70)	1		3.33	(1.07–9.87)	1	
Girls	2.35	0.98–5.53)	1.06	(0.21–5.36)	6.57	(3.92–10.81)	1.18	(0.44–3.19)	4.69	(2.54–8.52)	1.41	(0.40–5.01)
Course												
4th ESO	1.77	(0.57–5.38)	1		4.14	(1.98–8.45)	1		3.55	(1.59–7.69)	1	
2nd Baccalaureate	1.92	(0.47–7.40)	1.08	(0.18–6.39)	5.76	(2.61–12.28)	1.39	(0.48–4.04)	4.81	(2.01–11.06)	1.35	(0.42–4.33)
VET	6.66	(1.66–23.16)	3.76	(0.65–21.59)	20.0 *	(9.24–38.03)	4.83 *	(1.74–13.39)	6.66	(1.66–23.16)	1.88	(0.39–8.89)
Education level of parents												
University	2.50	(0.80–7.49)	1		3.33	(1.25–8.57)	1		2.5	(0.80–7.49)	1	
Secondary	0.97	(0.13–6.16)	1.43	(0.24–8.33)	4.85	(2.02–11.17)	3.21	(0.94–10.96)	5.82	(2.63–12.4)	1.43	(0.24–8.33)
Primary	3.57	(0.88–13.25)	0.39	(0.04–3.69)	10.71	(4.87–21.92)	1.46	(0.40–5.29)	3.57	(0.88–13.25)	2.33	(0.59–9.11)
SEP												
Lower SEP	3.81	(1.43–9.74)	1		6.66	(3.20–13.36)	1		5.71	(2.58–12.17)	1	
Medium SEP	1.73	(0.43–6.72)	0.46	(0.09–2.45)	5.21	(2.35–11.16)	0.78	(0.27–2.26)	4.31	(1.81–10.05)	0.76	(0.23–2.42)
Higher SEP	1.20	(0.16–8.11)	0.32	(0.04–2.79)	7.22	(3.27–15.21)	1.08	(0.38–3.11)	2.41	(0.60–9.17)	0.42	(0.09–2.04)
Municipality of residence												
Rural	1.70	(0.54–5.17)	1		4.54	(2.28–8.84)	1		3.97	(1.90–8.29)	1	
Urban	3.44	(1.29–8.86)	2.02	(0.46–8.89)	9.48	(5.31–16.35)	2.09	(0.86–5.04)	5.17	(2.33–11.07)	1.30	(0.45–3.78)
COVID-19 impact on the region												
More affected (Anoia)	1.85	(0.25–12.08)	1		7.41	(2.79–18.19)	1		5.55	(1.79–15.92)	1	
Less affected (others)	2.41	(1.08–5.27)	1.30	(0.16–10.63)	6.02	(3.65–9.76)	0.81	(0.28–2.36)	4.01	(2.16–7.31)	0.72	(0.21–2.54)

* *p* < 0.05. Abbreviatures: SEP = socioeconomic position. Terciles obtained from McArthur Scale results, which inquired for neighborhood self-perceived SEP. CIw: Cumulative Incidence of worsening of risky consumption for each substance between pre-COVID-19 and during COVID-19 confinement. ^a^ Worsening consumption: increasing or maintaining the prevalence of consumption of any substance studied during COVID-19 confinement.

## Data Availability

The data presented in this study are available upon reasonable request to the corresponding author. The data are not publicly available due to confidentiality reasons.
